# The Impacts of a Reading-to-Dog Programme on Attending and Reading of Nine Children with Autism Spectrum Disorders

**DOI:** 10.3390/ani9080491

**Published:** 2019-07-26

**Authors:** Stefania Uccheddu, Mariangela Albertini, Ludovica Pierantoni, Sara Fantino, Federica Pirrone

**Affiliations:** 1 Vet Ethology, Leemveldstraat 44, 3090 Overijse, Belgium; 2 Department of Veterinary Medicine, University of Milan, Via Celoria, 10, 20133 Milan, Italy; 3 CAN (Comportamento Animale Napoli) S.S.D.R.L., Via Camaldolilli, 79, 80128 Naples, Italy; 4 L’altra via, Psychology and Animal Assisted Intervention, Via Petrarca, 4, 09010 Pula, Italy

**Keywords:** dogs, children, Autism Spectrum Disorders, cognition, reading-to-dog programme

## Abstract

**Simple Summary:**

The purpose of this research was to compare reading motivation and attitude, as well as reading and cognitive skills, of school-age children diagnosed with Autism Spectrum Disorders (ASD) who attended a 10 session reading programme with and without the presence of a dog. Children who read to a dog had 100% attendance at sessions over the course of the programme versus 75% (range 25–100%) of children attending reading sessions without a dog. In addition, after the programme, they were significantly more motivated and willing to read at home, as perceived by their parents. However, there were no significant differences in scores on reading and cognitive tests either within each group or between groups. Based on these results, we can conclude that reading to a dog can have positive effects on an ASD child’s motivation and attitude toward reading. More research is needed to better understand if it can also have positive effects on children with ASD’s overall reading and cognitive abilities.

**Abstract:**

Poor knowledge is available on the effectiveness of reading to dogs in educational settings, particularly in children with Autism Spectrum Disorders (ASD). In this study, we test the hypothesis that reading to a dog improves propensity towards books and motivation to read after the end of the programme, as well as reading and cognitive skills in children with ASD. The study is a prospective, randomized controlled trial, consisting of testing and re-testing after a 10 sessions reading programme with and without the presence of a dog. Nine Children with ASD (6–11 years old) were randomly assigned to a control (CG, reading without a dog, n. 4) or experimental group (EG, reading to a dog, n. 5). Children’s attendance at reading sessions was recorded at each session. Parents’ perceptions were evaluated at the end of the programme to detect changes in children’s attitudes and motivation toward reading. Psychologist-administered validated reading (Cornoldi’s MT2 reading test; test of reading comprehension, TORC; metaphonological competence test, MCF) and cognitive tests (Wechsler intelligence scale for children Wisc IV, Vineland) to all children, at baseline and at the end of the reading programme. Compared with CG children, children in the EG group participated more frequently in the reading sessions, and they were reported to be more motivated readers at home after the programme. However, there were no differences on reading and cognitive tests’ scores either within each group of children or between groups. Further studies are warranted in order to understand whether and how incorporating dogs into a reading programme is beneficial to Children with ASD at the socio-emotional and cognitive level.

## 1. Introduction

Launched in 1999 by Intermountain Therapy Animals, the Reading Education Assistance Dogs (READ) is the first programme and still one of the most comprehensive involving animals to strengthen children reading skills [1]. The Reading Education Assistance Dogs^®^ programme improves children’s reading and communication skills by employing a powerful method: reading to a dog (R.E.A.D. webpage)

Recent work focused on children has shown that reading-to-dog programmes might reduce blood pressure and offer a nonjudgmental, safe environment in which to practice reading [2,3]. Dogs seem able to offer children a unique type of emotional support in the education setting because they are fully capable of being active, supportive listeners, but are also unable to verbally criticize or comment upon a child’s reading abilities [4]. In the wider literature [1], measurements of reading skills have included improved scores on test of reading comprehension (TORC), measures of academic progress (MAP), reading rate, and reading ability [1]. According to Pillow-Price et al. [5], all reading scores for children participating in a reading programme improved significantly. Sorin et al. [6] noted improvements in reading, behavior, confidence, self-esteem, and school attendance with special education students who worked on literacy skills with dogs. Changes in reading motivation may reflect a better reading performance [1]. In Guthrie and Cox [7], engaged and motivated children who opened a book more frequently were also highly achieving in reading abilities because cognitive functioning was powerfully facilitated through interest and motivation [8].

The presence of a dog has already been suggested to reduce physiological parameters of stress (decreased blood pressure [9] and cortisol [10]) in children with autism. A child with autism spectrum disorder improved on the Dynamic Indicators of Early Literacy Skills (DIBELS) and Elementary Reading Attitudes Scale (ERAS) after completing a reading-to-dog programme [11]. Based on the final version of DSM-5 [12], autism is currently counted in one general term, Autism Spectrum Disorders (ASD), with three different levels, level 1 (requiring support), level 2 (requiring substantial support), and level 3 (requiring very substantial support). ASD is characterized by delays in the development of multiple basic functioning including socialization and communication and behavioral challenges (such as rituals and repetitive behaviors) [12]. In the clinical setting, anxiety-related concerns are among the most common presenting problems for school-age children and adolescents with ASD [13]. Recently, one study developed an educational setting in which three Children with ASD read social stories in the presence of therapy dogs [14]. The authors aimed to test the hypothesis that the presence of a therapy dog improves the effectiveness of Social Story method, which is used to communicate clear and detailed information to autistic children on a context, skill, achievement, or concept [15]. Although improvements in these children’s indicators of social skills were reported (e.g., increased frequency of the initiations of social interactions and decreased level of prompt needed to provide the expected social response), interpretation of these indicators can be difficult, and the quality of the evidence is still unclear, also due to the small sample size used [1].

Here, we decided to apply recent and innovative psychological approaches to detect potential improvements in reading abilities and changes in behavioral and emotional processes in ASD school-age children’s reading in the presence of a dog compared with children reading without a dog. Moreover, we compared attendance at sessions of children and parents’ perception of their reading motivation and willingness to read. The main hypothesis was that a social environment enriched by the presence of a dog strengthens the effectiveness of a reading programme in enhancing both reading and cognitive abilities in Children with ASD.

## 2. Materials and Methods

### 2.1. Participants

A total of nine children in the age range of 6 to 11 years (mean 7 ± 0.45 SE), seven boys and two girls, were recruited from the CTR Esperienze ONLUS (Comunicazione Territorio Relazioni) Cagliari, Sardinia, Italy, where the reading sessions took place.

Informed consent was obtained from parents of all children, who were previously advised by the facility staff members of an experimenter’s presence for the videotaping procedure. In signing the consent, parents ensured that there was a clear understanding of the information given to them, and also that they agreed with that and with the disclosure of their personal details. Besides favorable opinion from a clinical psychologist or neuropsychiatry holding also a certification as Board Certified Behavior Analyst^®^ (BCBA^®^), to be eligible for participation in the programme, children were required to: (a) be diagnosed according to the diagnostic tools described in the DSM-5 and in the guidelines elaborated by the Italian Ministry of Health guidelines. The diagnosis was determined by a multidisciplinary equipe composed of a child neuropsychiatrist, a psychologist specialized in child development, and a pedagogist. The DSM-5 diagnosis also includes new guidelines for categorizing autism by level. There are three levels, each reflecting a different level of support each child needs (from level 1: little support, to level 3: higher support); (b) show lack of initiation of appropriate social response in a given social situation during therapy or free-time activities; (c) have some reading prerequisites, such as the ability to open and browse through a book; (d) be willing to interact with dogs, as evaluated in a preintervention screening; (e) possess basic speaking skills, and (f) immunocompetency. Fear of dogs was considered an exclusion criterion. Diagnosis and severity level have been established by the neuropsychiatry according to [12].

### 2.2. Reading Session

Children with ASD were randomly divided into two groups according to demographics characteristics and severity levels expressed in the diagnosis: (1) the experimental group (EG, n. 5: four boys and one girl; mean 7.60 ± 2.30 SE) read a book with a dog present, and (2) the control group (CG, n. 4: three boys and one girl; mean 8.25 ± 1.73 SE) read a book without a dog. Details on age, gender, and level of severity indicated in the diagnosis are reported in Table 1. Groups were homogeneous in terms of mean age and diagnosis. Both EG (experimental group) and CG (control group) were involved in 10 weekly group sessions, run over a period of 70 days. Each session was approximately 30 min in length, without pauses, during which children read a book one-on-one, upon request by the psychologist. A book was selected by the psychologist at the beginning of the programme. Both EG and CG children read the same book. A copy of the book was available for each child. The same psychologist was present for all the sessions for both EC and CG groups. The psychologist, before starting the reading session, reminded the children of the rule of the session. The rules were presented, if necessary, more times during the session, only in oral form (Now we are going to read to the dog. Her name is Bella/Lilli. The dog is pleased to listen to our reading, but we need to respect some specific rules: please, do not be loud, do not run, do not touch the dog since this is going to make the dog fearful. We are not going to pass through the benches during the reading session. We cannot touch the dog during the session, but we can talk with her).

Sessions were performed in the afternoon in order to exclude parental factors/obligations that could impact the child’s attendance.

As for the experimental group, two dogs (both neutered females, mixed-breed, 2 and 8 years old) participated in the sessions, one at a time, on alternate weeks. Two dogs were chosen by a team composed of two veterinarians expert in behavior and welfare and a psychologist specialized in animal-assisted intervention. Inclusion criteria considered their kindness and cooperation when handled by children, their interest in people, and absence of any signs of anxiety, fearfulness, reactivity, or aggression. The dogs, both neutered females, mixed-breed dogs, were 2 and 8 years old (mean 5.0 ± 3.0 SE) and weighed between 3 and 18 kg (mean 10.5 ± 7.5 SE) at the time of the sampling period. Dogs were recruited from the local nonprofit organization “Effetto Palla ONLUS”, with the aim of enhancing their socialization and adoption rates [16]. Dogs were subjected to regular health screening and behavioral monitoring by a veterinarian with expertise in animal behavior and welfare. In order to be eligible for participation in the reading programme, the dogs were required to be in perfect clinical health (i.e., free from pain, external and internal parasites, and immunized). These dogs’ characteristics, behavior, and welfare during the reading sessions have been described also in more detailed in [17]. Child–animal interaction was limited to verbal contact: no child-initiated contacts with dogs were allowed. Children could only talk to the dog, and they did so by praising her or asking whether she enjoyed the story or was getting bored.

### 2.3. Setting Room

The two dogs were handled by a female veterinarian expert in animal welfare and behavior, who was familiar with them and was always present during the sessions to guarantee their well-being.

Sessions were performed in a 6 × 5 m carpeted room at the facility, where children were also involved in other activities, in the presence of a psychologist. In more detail, at reading sessions, one visiting dog, one dog handler/veterinarian, one psychologist, and one experimenter were always present. The room temperature ranged between 20 °C and 24 °C. Two 30 cm high benches were placed to separate the room into two identical spaces, one for the dog and one for the children [10].

### 2.4. Test

At baseline (T0) and at the end of the 10 sessions programme (T1), the psychologist administered validated reading and cognitive tests to all the children (Table 2). Pre- and posttest in both groups followed the same order. Reading tests such as Cornoldi reading test (MT2) [18], test of reading comprehension (TORC) [19], metaphonological competence (MCF) [20] and cognitive tests, Wechsler intelligence scale for children (Wisc IV) [21], and Vineland [22].

Session attendance was recorded in both groups. A short self-report questionnaire was prepared by the psychologist by reviewing similar literature [23] in order to collect parents perceptions after the 10 reading sessions. The questionnaire, presented in Table 3, was composed of seven yes/no closed questions. The questions focused on the perception of the parents about: (1) reading motivation, (2) motivation to follow the programme, (3) social skills, and (4) attention towards dogs.

### 2.5. Statistical Analysis

Data was analyzed with SPSS, version 25.0 (SPSS Inc, Chicago, IL, USA) through nonparametric statistics as they did not follow a normal distribution (Shapiro–Wilk normality tests, all *p* > 0.05). The Mann–Whitney U test was used to compare differences between the two groups, while the Wilcoxon signed-rank test was used for paired data. Due to the multiple comparisons, Benjamini–Hochberg multiple testing correction [24] was applied. Fisher’s exact test was used to investigate associations between the presence of the dog and both children’s attendance and parents’ answers in the questionnaire. Values of *p* < 0.05 were considered statistically significant.

## 3. Results

### 3.1. Session Attendance

**Figure 1:**
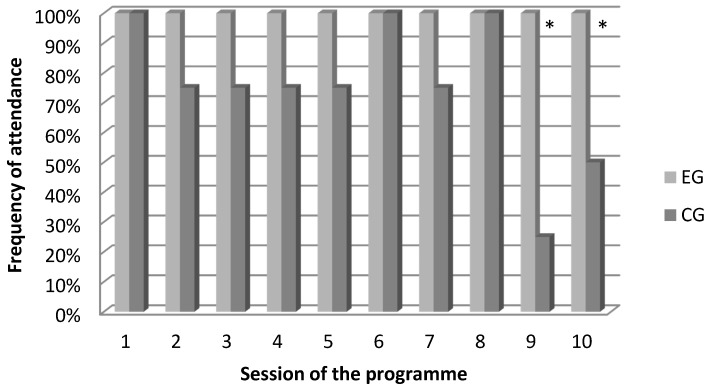
Children’s attendance. EG and CG children. EG = Experimental group; CG = Control Group; * p < 0.05.

EG children achieved 100% attendance in each reading session, which was statistically higher than the 75% of CG children (range 25–100%, U = 11.0, z = −3.468, *p* = 0.002, Figure 1). In particular, in CG children, attendance was significantly different on day 9 (Fisher’s exact test *p* = 0.04) and day 10 (Fisher’s exact test *p* = 0.05) compared with the other days.

### 3.2. Reading Tests

We explored the two domains of MT2, namely speed (S) and accuracy (A), the reading comprehension (RC) for the TOR test, and the five domains [18] for the MCF test: Recognition (RE) Fluidity (F), Phonemic (FO), Segmentation (SG), Letter deletion (LD). On all reading tests, no significant differences were found between T0 and T1 within each group (Mann–Whitney U test, *p* > 0.05) or even between the EG and CG groups at each time point (Wilcoxon signed-rank test, *p* > 0.05) (Table 4 and Table 5).

### 3.3. Cognitive Test: WISC IV Test and Vineland Tests

The five domains of the WISC IV test have been explored: Intelligence Quotient (IQ), Fluid Reasoning Index (RF), Processing Speed Index (PS), Verbal Comprehension Index (VC), Working Memory Index (WM). Vineland test’s domains have been analyzed: Compressive Results (CO), Communication (CM), Daily Living Skills (DLS), Socialization (S), and Motor Skills (MS).

On all the cognitive tests, no significant differences were found between T0 and T1 within each group (Mann–Whitney U test, *p* > 0.05), as well as between the EG and CG groups at each time point (Wilcoxon signed-rank test, *p* > 0.05) (Table 6 and Table 7).

### 3.4. Parents’ Questionnaire

Questions and answers reported by the parents of the EG and CG children are shown in Table 3.

## 4. Discussion

In the present study, we evaluated a programme that aims to understand the impact of 10 weekly reading sessions with dogs on children with ASD to read. Attendance and parents’ perceptions were evaluated. Validated reading and social tests were employed prior to the beginning and after the end of the programme in order to offer an evidence-based evaluation approach. To our knowledge, this is the first time that tests measuring reading and social skills have been applied to assess the effectiveness of a reading-to-dog programme in children with ASD. Being willing to interact with dogs has been considered as inclusion criteria: this makes it difficult to generalize the results to all children with ASD, although it might be applicable to other children with ASD who happen to like dogs (or at least not dislike them). However, this study wants to work as a pilot in the reading-to-dog programme field.

Motivation has been defined as “a psychological process in which personality traits (e.g., motives, reasons, skills, interests, expectations, and future perspectives) interact with perceived environmental characteristics” [25]. Thus, student motivation can be affected by changes in their learning environment. In our study, the reading-to-dog programme significantly increased the propensity of children to read at home and look autonomously for a book, as showed by EG parents scoring higher on the related questions of the survey compared with CG parents immediately after the end of the programme. This is in line with what is reported in reading studies [1], in which motivation is often discussed in terms of intrinsic motivation (motivated from internal factors; e.g., curiosity to read, enjoyment of the experience) and extrinsic (motivated by external factors; e.g., to get a good grade). Children in the EG group were also perceived by their parents as having a significantly higher motivation to follow reading sessions. EG children actually attended the sessions significantly more frequently (100% attendance) than those in the control group (25% to 100% attendance). According to Newman-Ford et al. [26] attendance is a measure of a student’s motivation for learning, which is considered a galvanizing energy in the learning process. From this perspective, it is not surprising that EG children were significantly more motivated to do homework at home than controls, as reported by their parents. The dog might have acted as motivator for children to attend, which might be due to a dog’s recognized ability to be an active, nonjudgmental listener [1]. As reported in [27], “The dogs ‘listened’ while the students were reading at their own pace. The dogs did not laugh, judge or criticize them, and therefore they were not embarrassed by their own mistakes”. Moreover, in [28]), children with autism interacted most frequently and for the longest periods with a real dog in comparison with objects or a person. The presence of the dog assumed an important role during the session. The authors concluded that students reading in the presence of a dog were more likely to participate in reading-to-dog sessions, because that was an environment in which they could build their self-confidence [27]. Also. children with pervasive developmental disorders (including autism) were more playful in interaction with a live dog compared with toys, and also more aware of their social environment in the presence of the dog [29]. However, in the questionnaire, when parents were asked to answer to a specific question about “attention to dogs”, no differences between EG and CG were reported. We wanted to ask this question in order to understand if the children with ASD were more aware of the social environment, as reported in literature [29].

In our study, children’s engagement in social interactions with peers was not increased at the end of the programme in both groups, according to parents’ perceptions. Similarly, Socialization Area results obtained on Vineland tests (for example, the score related to Plays with peer/s for 5 min under supervision, Plays with peer/s for 20 min under supervision, Asks others to play or spend time together) showed no improvements in social skills of children from both groups when the programme was over. This is in contrast with what was reported in the study by Grigore et al., [14], in which the author found improved social interactions in three preschool autistic children following a combined social story method and canine-assisted intervention. As far as we know, there are no other published researches conducted with children with ASD reporting results based on engagement in social interactions with peers. Paul and Serpell [30] found that normal families who obtained a dog, 1 month later engaged in more leisure activities together and their children were more often visited by friends. In a classroom of first-graders, the presence of a dog led to a better social integration among students, as documented via indirect psychometric indicators [31] as well as via direct behavior observation [32].

The possible role of the Oxytocin (OT) in these child–dog interactions during reading-to-dog sessions needs to be underlined too. Nagasawa et al. [33] assessed the effect of 30 min of interaction between dogs and their owners, particularly the duration of friendly gazes from the dogs to the owners. In a control condition lasting for 30 min, owners were instructed not to look at their dogs directly. In the normal interaction condition, longer gaze was linked to higher OT levels in the owner, while this was not the case in the control condition without eye contact. The interaction, even without direct contact, is related to OT increases that are strictly related to social interaction (see [34] for a detailed review). The release of OT via contact with animals may contribute to explain many of the effects of dog–human interactions.

As for both groups, we found no significant gains in children’s reading test (MT2, TOR, MCF) scores after taking part in our reading-to-dog programme. In contrast, Konarski et al. [11] reported improved Dynamic Indicators of Early Literacy Skills (DIBELS) and Elementary Reading Attitudes Scale (ERAS) in a child with autism spectrum disorder after completing a reading-to-dog intervention. However, this was a case study, which did not use any control measures or include a case series, and therefore it does not allow to conclude that any change observed is due to the intervention being studied rather than to other factors. Several other authors described positive effects of reading-to-dog programmes in children (see [1] for review). For example, Fisher et al. [35] applied the Neale Analysis of Reading Ability [36] to test reading abilities in one child, before and after participating in a BaRK programme. BaRK is a free programme that involves reluctant readers in the middle-upper primary school classes. In this programme, a child was involved in eight weekly reading sessions with a dog. The results indicated a dramatic improvement between pretest and post-test scores for both reading accuracy and comprehension, with greater gains being made in comprehension skills. In [37], 26 children had higher scores after reading to a dog on the Gray Oral Reading Test (GORT-4), in which the child has to read aloud narrative passages (of medium length) and, for each passage, answer to multiple-choice comprehension questions read by the examiner. These results were supported by those collected by The Intermountain Therapy Animal [38] that indicated students’ reading skills improved by two to four grade levels during a reading programme. However, again, failure to use appropriate controls makes it impossible to draw meaningful conclusions from these studies. Booten [39] and Petersen [40] included a control group in their investigation, and they did not report any differences between children who read to a dog and those who read without a dog. Conversely, Treat et al. [41] found improved reading fluency, accuracy, and comprehension after reading to a dog, while in the study by LeRoux et al. [27] children in the dog group scored higher on the Neale reading comprehension test compared with the control groups, and Kirnan [42] found an improvement in reading skills based on teachers perception. It should be noted that all these studies involved typical children, making it difficult to compare results with ours. In fact, a meta-analysis by Fuchs [43] revealed that the reading achievement of students with a learning disability is significantly different from that of typical students, even if low-achieving students are considered: students with learning disabilities have more severe reading problems than others [43]. Overall, children with ASD can be characterized by a triad of persistent impairments with core deficits in social interaction, language, and communication, as well as restrictive, repetitive thoughts, routines, and behavior patterns: ASD and learning disability are then co-associated. ASD is more likely to be present in individuals with a learning disability, impacting on all aspects of learning, especially among more severely affected individuals [44]. In our study, children with ASD had to follow important but easy rules related to the setting. The reason for this limitation is dual. As for dogs, this allowed activities to be predictable and controllable [17,45]. For children, it was a way to receive a simple but useful rule.

There are some limitations to our study, so the findings should be interpreted with caution. First, the programme involved a small sample size and did not control for the confounding effect of variables, including parenting styles but also comorbid outcomes such as anxiety, which makes it difficult to generalise to a wide population. Second, although parent-completed questionnaires are considered as accurate as developmental screening instruments (see [46] for example), parents were required to interpret their children’s motivation and attitudes, inevitably resulting in a degree of subjectivity. In addition, it is possible that the parents’ answers were influenced by perceptions of which answers would be deemed acceptable, even if the questionnaire was anonymous. Third, we implemented a short-term intervention, and future studies should examine interventions over a longer time (e.g., the entire school year), possibly analyzing academic performances. However, a standard programme for Children with ASD has not been developed and validated yet [42].

## 5. Conclusions

In conclusion, reading to a dog has the potential to bring significant improvements to typical children’s social and reading abilities [1]. The results of the present pilot study suggest that such a programme can have specific effects on session attendance and literacy motivation at home in children with ASD, as perceived by their parents. Previous research demonstrated that increased engagement in reading is linked to improved academic performance [47,48]. Thus, the attendance at (and engagement in) reading sessions, enriched by the presence of a dog should be further examined, together with the critical aspects of literacy, including testing accuracy, fluency, and comprehension.

The spatial setting used in this pilot can be applied in following studies in order to create a perfect welfare area for dogs and to take the chance to teach a rule to the children with ASD. In order to evaluate the success of a reading-to-a-dog programme, not only validated tests but also percentage of attendance and parents’ perceptions should be taken into account. The next step should include large-scale, randomized control trials with longitudinal examinations of effects, to provide more tangible and reliable findings not only for children with ASD but also for dogs.

A recent review [1] reported positive results based on implementation of a reading-to-dog programme. Unfortunately, these are mostly based on ad-hoc reports, without undergoing a peer-review process [42]. These studies did not randomly allocate children to intervention or control groups, and only small groups (or case study) were investigated. Although extensive generalization should be avoided, the results of our study provide some tentative support for the effectiveness of a reading-to-dog programme based on the use of objective assessments. Specific tests did not confirm any effect on children’s social and literacy skills due to the presence of a dog. More research is therefore needed to understand the impact of this type of intervention, considering potential confounding variables, including individual factors or a different number of sessions.

## Figures and Tables

**Table 1:** Age, gender, and diagnosis of Children with ASD involved in the project.

Experimental Group			Control Group		
Age (Years)	Gender	Severity Level ^1^	Age (Years)	Gender	Severity Level ^1^
11	M	1	7	M	1
9	M	1	10	M	1
6	M	2	10	F	1
6	F	1	6	M	2
6	M	1	-	-	

M = Male; F = Female; ASD = Autistic spectrum disorder. ^1^ According to [12].

**Table 2:** Different tests administered to children by psychologist at T0 and T1.

Area of Interest	Test	Details	Domains	Items	Scores
Reading	Cornoldi Reading Test (MT2) [18]	assesses reading literacy	Fluency (speed and accuracy)	2	Speed: syllabus in one second;Accuracy: number of auto-correction
Reading	Test of Reading Comprehension (TORC) [19]	measures a child’s abilities in reading comprehension	Reading comprehension	1	0 to 100 (0 low level, 100 higher level)
Reading	Metaphonological Competence (MCF) [20]	measures the child ability to talk about a topic and explain his or her use or understanding of the phonological awareness skill	Recognition, Fluidity, Phonemic, Segmentation, Letter deletion, Final deletion	5	0 to 100 (0 low level, 100 higher level)
Cognitive	Wechsler Intelligence Scale for Children (Wisc IV) [21]	measures a child’s abilities in some cognitive domains. It generates a Full-Scale IQ (formerly known as an intelligence quotient or IQ score) that represents a child’s general intellectual ability	Intelligence Quotient, Fluid Reasoning Index, Processing Speed Index, Verbal Comprehension Index, Working Memory Index	5	70 to 130 (70 low level, 130 higher level)
Adaptive behavior	Vineland [22]	measures the personal and social skills of individuals from birth through adulthood	Compressive Results, Communication, Daily Living Skills, Socialization, and Motor Skills	5	Specific for the age range: 34 to 144 (34 low level, 144 higher level)

**Table 3:** Parent-completed questionnaire.

Questions	EG Parents Answers		CG Parents Answers		*p* (Fisher’s Exact Test)
At the End of the Reading Programme	Yes	No	Yes	No	
(1) Was the child pleased to read?	4	1	1	3	*p* > 0.05
(2) Was the child looking for any book autonomously or in presence of an adult?	4	1	0	4	*p* = 0.04
(3) Was the child more motivated and enthusiastic to read a book?	4	1	0	4	*p* = 0.04
(4) Was the child able to pay more attention to dogs in daily routine?	4	1	2	2	*p* > 0.05
(5) Was the child able to keep a relationship with other children in the group?	1	4	1	3	*p* > 0.05
(6) Was the child more motivated in doing homework at home?	4	1	0	4	*p* = 0.05
(7) Was the child motivated to follow the sessions?	4	1	2	2	*p* > 0.05

EG = Experimental group; CG = Control group.

**Table 4:** Statistical results of the MT2 and TOR tests at baseline and at the end of the reading sessions (*p* > 0.05). Mean ± Standard Deviation is reported.

Group	Time Points	MT2-S	MT2-A	TOR-RC
Experimental	T0	1.5 ± 1.2	7.3 ± 2.3	53.5 ± 12.0
	T1	1.7 ± 1.5	7.3 ± 2.3	64.0 ± 26.8
Control	T0	2.7 ± 0.4	8.3 ± 2.9	76.5 ± 33.2
	T1	3.0 ± 0.3	8.3 ± 2.8	72.3 ± 24.5

MT2-S = Cornoldi reading test speed; MT2-A = Cornoldi reading test accuracy; TOR-RC = TOR test reading comprehension.

**Table 5:** Statistical results of the CMF tests at baseline and at the end of the reading sessions (*p* > 0.05). Mean ± Standard Deviation is reported.

Group	Time Points	RE	F	FO	SG	LD
Experimental	T0	30.0 ± 28.2	33.3 ± 14.4	35.0 ± 25.9	37.5 ± 17.6	21.2 ± 21.3
	T1	26.6 ± 22.5	28.3 ± 20.2	35.0 ± 25.9	35.0 ± 25.9	35.0 ± 2.9
Control	T0	30.0 ± 28.8	50.0 ± 0.0	27.5 ± 31.8	27.5 ± 31.8	27.5 ± 31.8
	T1	50.0 ± 0.0	50.0 ± 0.0	30.0 ± 28.2	30.0 ± 28.2	50.0 ± 0.0

RE = Recognition; F = Fluidity; FO = Phonemic; SG = Segmentation; LD = Letter deletion.

**Table 6:** Statistical results of the WISC tests at baseline and the end of the reading sessions (*p* > 0.05). Mean ± Standard Deviation is reported.

Group	Time Points	IQ	RF	PS	VC	WM
Experimental	T0	75.2 ± 16.4	84.0 ± 18.7	79.0 ± 13.34	84.7 ± 5.6	75.5 ± 16.2
	T1	75.0 ± 16.6	82.5 ± 20.2	80.5 ± 11.4	84.7 ± 5.6	74.0 ± 17.4
Control	T0	108.2 ± 24.7	86.0 ± 33.1	106.5 ± 15.7	102.0 ± 26.9	133.0 ± 0.0
	T1	100.0 ± 25.2	83.0 ± 35.5	103.7 ± 10.3	96.0 ± 21.7	92.5 ± 44.5

IQ = Intelligence Quotient; RF = Fluid Reasoning Index; PS = Processing Speed Index; VC = Verbal Comprehension Index; WM = Working Memory Index.

**Table 7:** Statistical results of the Vineland tests at baseline and the end of the reading sessions (*p* > 0.05). Mean ± Standard Deviation is reported.

Group	Time Points	CO	CM	DLS	S	MS
Experimental	T0	53.7 ± 19.6	69.2 ± 25.8	45.0 ± 8.3	50.0 ± 17.1	46.5 ± 9.1
	T1	76.3 ± 29.2	97.0 ± 36.7	76.3 ± 29.6	62.6 ± 22.1	48.0 ± 0.0
Control	T0	63.4 ± 26.1	74.8 ± 29.8	50.4 ± 10.7	55.0 ± 19.0	40.0 ± 0.0
	T1	78.5 ± 34.6	99.0 ± 45.2	78.0 ± 36.8	65.5 ± 21.9	55.0 ± 0.0

CO = Compressive Results; CM = Communication; DLS = Daily Living Skills; S = Socialization; MS = Motor Skills.
